# Supplementary material for: AMPK-Dependent YAP Inhibition Mediates the Protective Effect of Metformin against Obesity-Associated Endothelial Dysfunction and Inflammation
Source: Antioxidants (Basel). 2023 Aug 28;12(9):1681. doi: 10.3390/antiox12091681 (PMC10525300; doi:10.3390/antiox12091681)
Supplement: Supplementary file 1 [file antioxidants-12-01681-s001.zip › antioxidants-2545010-supplementary.pdf]

antioxidants

MDPI

Supplementary Materials

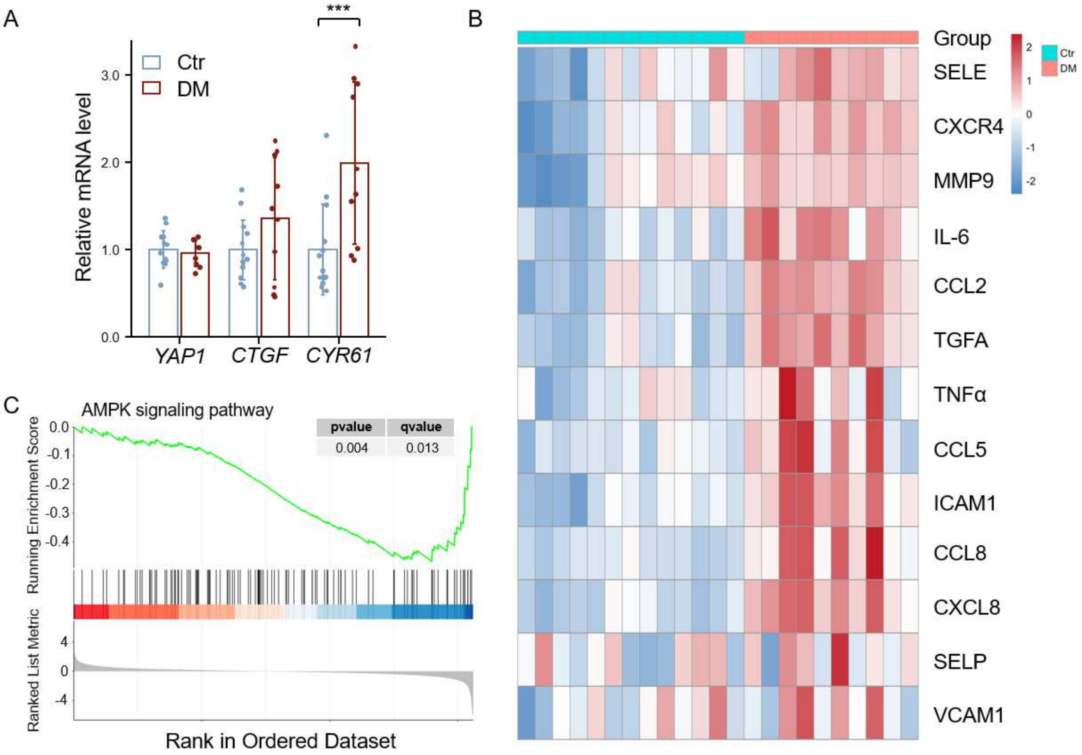

Figure S1. The expression of YAP signaling in diabetic coronary artery lesions. (**A**) The relative expression level of YAP target genes (CTGF and CYR61) in diabetic-hypercholesterolemic pigs (DM) compared with hypercholesterolemic pigs (Ctr). (**B**) Differential expression heatmap of vascular inflammatory factors including chemokines, adhesion molecules, cytokines and formins. (**C**) GSEA result of KEGG pathway: AMPK signaling pathway downregulated in diabetic group. GSEA: Gene set enrichment analysis; KEGG: Kyoto Encyclopedia of Genes and Genomes. Data are means ± SD. *** $p < 0.001$.

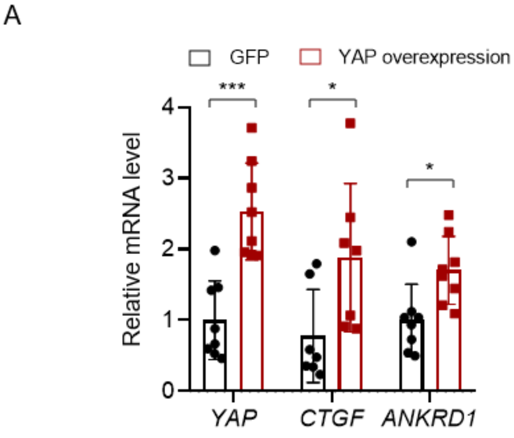

Figure S2. Expression efficiency of YAP adenovirus in isolated aortic rings. **(A)** Quantification of the mRNA expression of YAP and target genes (CTGF and ANKRD1) in isolated aortic rings infected by YAP adenovirus. Data are means ± SD. * $p < 0.05$, *** $p < 0.001$.

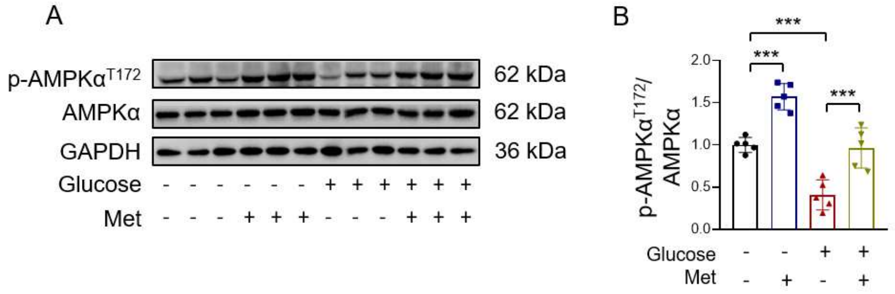

Figure S3. Metformin reverses high glucose-induced inhibition of AMPK phosphorylation in HUVECs. (**A, B**) Representative immunoblots (**A**) and quantification of AMPK phosphorylation (**B**) in HUVECs treated with 30 mM glucose and 10 mM metformin (Met). Data are means ± SD. *** $p < 0.001$.

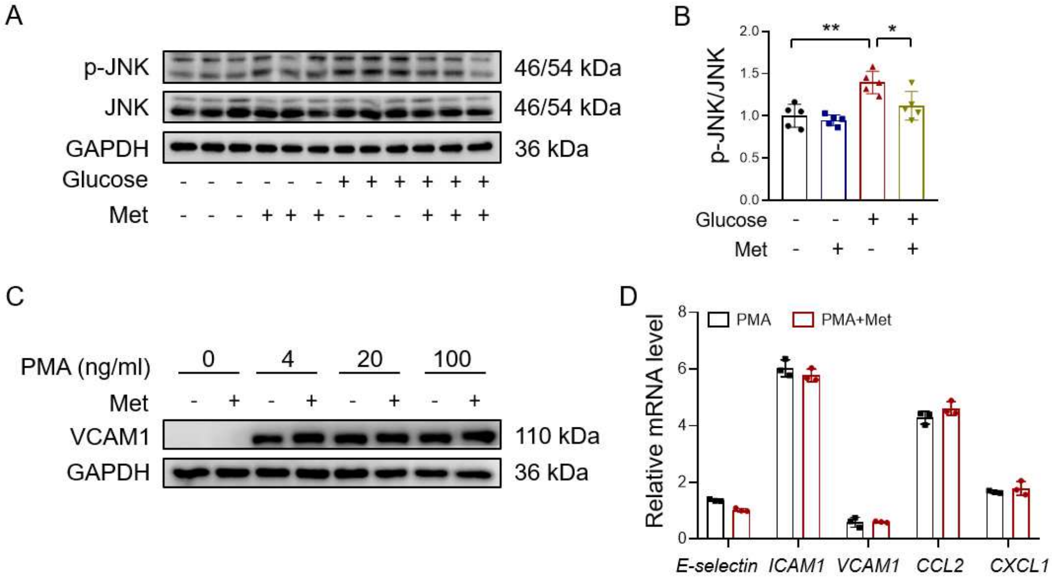

Figure S4. Metformin reduces vascular inflammation through YAP-JNK pathway. (**A, B**) Representative immunoblots (**A**) and quantification of JNK phosphorylation (**B**) in HUVECs treated with 30 mM glucose and 10 mM metformin (Met). (**C**) Representative immunoblots of VCAM1 and GAPDH in HUVECs treated with metformin and different concentrations of JNK stimulator PMA. (**D**) Quantification of the mRNA expression of pro-inflammatory genes (E-selectin, ICAM-1, VCAM-1, CCL2 and CXCL1) in HUVECs treated with PMA and metformin. Data are means ± SD. * $p < 0.05$, ** $p < 0.01$.
